# *In Vitro* and *In Vivo* Assessments of Cardiovascular Effects with Omadacycline

**DOI:** 10.1128/AAC.00320-16

**Published:** 2016-08-22

**Authors:** S. Ken Tanaka, Stephen Villano

**Affiliations:** Paratek Pharmaceuticals, Boston, Massachusetts, USA

## Abstract

Omadacycline is a first-in-class aminomethylcycline antibiotic with a broad spectrum of activity against Gram-positive and Gram-negative aerobes and anaerobes and atypical bacterial pathogens. A series of nonclinical studies, including mammalian pharmacologic receptor binding studies, human ether-a-go-go-related gene (hERG) channel binding studies, studies of the effects on *ex vivo* sinoatrial (SA) node activity, and studies of *in vivo* effects on cardiovascular function in the cynomolgus monkey, was undertaken to assess the cardiovascular risk potential. Omadacycline was found to bind almost exclusively to the muscarinic subtype 2 acetylcholine receptor (M_2_), and in the SA node model it antagonized the effect of a pan-muscarinic agonist (carbamylcholine) in a concentration-dependent manner. Omadacycline exhibited no effect on hERG channel activity at 100 μg/ml (179.5 μM), with a 25% inhibitory concentration of 166 μg/ml (298.0 μM). Omadacycline had no effect on QTc in conscious monkeys at doses up to 40 mg/kg of body weight. Overall, omadacycline appears to attenuate the parasympathetic influence on the heart rate but has a low potential to induce cardiac arrhythmia or to have clinically significant cardiovascular toxicity.

## INTRODUCTION

Omadacycline is a first-in-class aminomethylcycline antibiotic (1) with broad-spectrum microbiological activity, including potent activity against Gram-positive and atypical bacteria and good activity against Gram-negative and anaerobic bacteria (2 – 4). Results from clinical studies indicate that omadacycline can be administered as once daily oral or intravenous (i.v.) therapy; the bioavailability of the oral tablet formulation currently in use is 35% (5, 6). Clinical development is proceeding to evaluate omadacycline for the treatment of serious infections, including community-acquired bacterial pneumonia (CABP) and acute bacterial skin and skin structure infections (ABSSSI).

An important component of the development of novel pharmacologic compounds is an evaluation of the potential for cardiovascular toxicity (7). A variety of drugs, including those belonging to the macrolide, ketolide, and fluoroquinolone classes of antibiotics, are well-known to block the cardiac potassium channel encoded by the human ether-a-go-go-related gene (hERG), which causes prolonged cardiac repolarization with the attendant potential for an increased risk for ventricular arrhythmias (8 – 14).

A series of nonclinical *in vitro* and *in vivo* studies was undertaken with omadacycline with the objective of evaluating the potential for cardiovascular toxicity. These studies investigated the affinity of omadacycline binding to mammalian pharmacologic receptors, binding activity on hERG channels, *ex vivo* effects on the sinoatrial (SA) node, and *in vivo* effects on cardiovascular function and physiology in the cynomolgus monkey.

## MATERIALS AND METHODS

### Receptor binding.

Standard radiolabeled ligand binding assays were performed with omadacycline at a concentration of 10 μM (5.57 μg/ml) and 59 pharmacologically relevant targets obtained from various sources, including human, rat, mouse, guinea pig, rabbit, and hamster tissues (LeadProfilingScreen; MDS Pharma, Bothel, WA). The percent inhibition of binding of the radiolabeled ligand was obtained from the average of two experiments performed in duplicate.

### hERG tail current in HEK293 cells.

The effects of omadacycline on the hERG tail current were recorded from human embryonic kidney (HEK293) cells stably transfected with hERG cDNA (Inveresk Research International, Ltd., Tranent, United Kingdom). Omadacycline was formulated in bath solution, shaken, and sonicated to give final perfusion concentrations of 100, 250, 500, and 1,000 μg/ml. A corresponding vehicle concentration of 100% bath solution was maintained in all perfusion solutions. Cells were transferred to the recording chamber and continuously perfused (at approximately 1 to 2 ml/min) with bath solution at room temperature. The effects of omadacycline or vehicle were investigated in four individual cells. The effect of 100 nM E-4031 (a K^+^ channel blocker) was investigated in two vehicle-treated cells to confirm the sensitivity of the test system to an agent known to block the hERG current. To establish an effect of omadacycline at each concentration, the magnitude of the steady-state tail current was compared to the value obtained under control conditions. The residual current (as a percentage of that for the control) was calculated for each treatment. To calculate the 25% inhibitory concentration (IC_25_), tail currents in the presence of omadacycline were first corrected for the mean vehicle rundown observed using a normalization process. The concentration-response relationship was plotted, and the IC_25_ value was calculated from a standard point-to-point fit to the data. In order to determine the highest concentration of omadacycline where no statistically significant difference in the result from that for vehicle-treated cells was observed, the values for 100, 250, 500, and 1,000 μg/ml omadacycline and 100% bath solution were pooled.

### *Ex vivo* SA node.

In a research protocol approved by the department of Preclinical Safety Europe, Novartis Pharma AG (Basel, Switzerland), New Zealand White female rabbits (*n* = 6) were sacrificed, and the heart was removed and placed in Tyrode's solution. The right atrium was separated from the rest of the heart. The preparations were mounted in a tissue bath and kept at 37 ± 0.5°C for at least 1 h for stabilization, during which the sinoatrial (SA) pacemaker activity was stable for at least 20 min. Action potentials (AP) were recorded intracellularly with a standard glass microelectrode (resistance, 10 to 70 MΩ) filled with 3 M potassium chloride (KCl) and connected to a high-input impedance-neutralizing amplifier (VF-180 microelectrode amplifier). Following stabilization, omadacycline was added to the Tyrode's solution of individual SA node preparations (*n* = 6) at effective concentrations of 0 (control), 0.05, 0.126, 0.422, 1.5, and 5 mM for 30 min. High-pressure liquid chromatography (HPLC) analyses before and after passage into the perfusion system were performed to measure the exact concentrations tested.

The following parameters were measured: the resting membrane potential (RMP); the maximum rate of depolarization (*V*_max_); the action potential amplitude (APA); the action potential duration at 50% (APD_50_) and at 90% (APD_90_) of repolarization; and the spontaneous beating rate (i.e., frequency), a characteristic of the pacemaker SA node. Electrophysiological measurements were assessed at the end of the 30-min perfusion period. The SA spontaneous frequency was evaluated by counting the number of beats within a 10-s period and then again within a 30-s period to express the results in number of beats per minute (bpm).

### Cardiovascular effects in conscious male cynomolgus monkeys.

A study evaluated the effects of omadacycline on cardiovascular function in conscious male cynomolgus monkeys (Battelle, Columbus, OH). The research protocol was approved by the Battelle Institutional Animal Care and Use Committee. Four nonnaive male animals were surgically implanted with a radiotelemetry transmitter, which collected electrocardiogram (ECG), systemic arterial blood pressure, and body temperature data. A pressure catheter was surgically implanted into the femoral artery, and two ECG leads were placed beneath the skeletal musculature cranial and caudal to the heart to emulate a standard lead II electrocardiogram. Animals were given either vehicle (PlasmaLyte A electrolyte solution for injection) or omadacycline at 5, 20, and 40 mg/kg of body weight according to a balanced Latin-square crossover design through a 22-gauge 1-in. catheter placed in the right or left saphenous vein. Systolic, diastolic, and mean blood pressures, heart rate, and body temperature data were collected at least 2 h prior to each dosing and for at least 24 h following the start of dosing on days 1, 8, 15, and 22. On each dosing day, at least 120 min of baseline ECG data were collected, and they continued to be collected throughout the dosing period through at least 5 h following the start of each dose administration. ECG waveform tracings of approximately 32 s in duration were produced on each day of dose administration (days 1, 8, 15, and 22) at a time prior to dosing; at approximately 0.25, 0.5, 1, 2, 4, and 5 h following the initiation of dosing while the animals were in an isolation box; and at approximately 8, 12, and 24 h following the initiation of dosing while they were caged. ECG interval measurements (RR, PR, QRS, and QT) were made on the ECG waveform tracings produced at each of the time points designated above. Rate-corrected QT (referred to as QTc or QTcF) intervals were calculated for each animal using the rate correction method of Fridericia, which involved dividing the QT interval by the cubed root of the RR interval.

### Statistical analysis.

For stable cells transfected with hERG, group mean values and the corresponding standard deviation (SD) or standard error of the mean (SEM) were reported. Statistical analysis was performed either by one-way analysis of variance (ANOVA) followed by Dunnett's or Bonferroni's multiple-comparison *post hoc* tests or by application of Student's *t* test for paired data. For hERG tail current studies, the groups were compared using one-way ANOVA followed by Dunnett's *t* test. For the SA node, ANOVA with repeated measurements followed by Dunnett's *t* test or with ordinary measurements followed by Dunnett's *t* test was used for the comparisons. For all experiments, differences were considered statistically significant at a *P* value of <0.05. Statistical analysis and graphical presentation of the data were done with GraphPad Prism (version 2.1 or 3.0) software (GraphPad Software, San Diego, CA, USA) or version 8 (release 8.2) of the SAS system.

## RESULTS

### Receptor binding.

Omadacycline (10 μM, 5.57 μg/ml) inhibited the ligand binding activity of one subtype of muscarinic acetylcholine receptor (M_2_) by 82%; there was no substantial effect on muscarinic receptor M_3_ or nicotinic acetylcholine receptors. Omadacycline did not inhibit or stimulate the ligand binding activity of adrenergic receptors or any other receptor target by more than 20% (Table 1). Further evaluation of muscarinic receptor interactions indicated that the concentration required for 50% inhibition (IC_50_) of M_2_ binding was 4.2 to 4.3 μM, whereas the IC_50_ for M_1_ binding was 29 μM and the IC_50_s for M_3_, M_4_, and M_5_ binding were >30 μM (Table 2). The interaction with M_2_ was weakly antagonistic (IC_50_, 21 to 28 μM) and not agonistic (concentration required for 50% of the maximum effect [EC_50_], >30 μM).

**TABLE 1:** Target radiolabeled ligands inhibited or stimulated by ≥10% by 10 μM (5.57 μg/ml) omadacycline

Target	Source	% inhibition or stimulation^*a*^
Adrenergic α_1B_	Rat	15
Adrenergic α_1D_	Human	14
Adrenergic β_1_	Human	11
Adrenergic β_2_	Human	−12
Bradykinin β_1_	Human	11
Calcium channel L type, benzothiazepine	Rat	12
Calcium channel L type, dihydropyridine	Rat	18
Dopamine D_3_	Human	12
GABAa, agonist site	Rat	14
Glutamate, kainite	Rat	14
Glutamate, *N*-methyl-d-aspartate, glycine	Rat	14
Histamine H_2_	Guinea pig	19
M_2_	Human	82
M_3_	Human	13
Neuropeptide Y_1_	Human	11
Nicotinic acetylcholine, central	Rat	−18
Phorbol ester	Mouse	14
5-Hydroxytriptamine, 5-HT_1A_	Human	10
Sodium channel, site 2	Rat	19

a Positive values represent inhibition, and negative values represent stimulation.

**TABLE 2:** Muscarinic receptor specificity of omadacycline

Target	Test no.^*a*^	EC_50_ or IC_50_ (μM)
M_1_ binding	1	29
M_2_ binding	1	4.3
M_2_ binding	2	4.2
M_2_ agonist	1	>30
M_2_ agonist	2	>30
M_2_ agonist	3	>30
M_2_ agonist	4	>30
M_2_ agonist	5	>30
M_2_ antagonist	1	21
M_2_ antagonist	2	27
M_2_ antagonist	3	22
M_2_ antagonist	4	28
M_3_ binding	1	>30
M_4_ binding	1	>30
M_4_ binding	2	>30
M_4_ binding	3	>30
M_4_ binding	4	>30
M_5_ binding	1	>30
M_5_ binding	2	>30
M_5_ binding	3	>30
M_5_ binding	4	>30

a The results of separate experiments using human recombinant receptors are shown.

### hERG tail current in HEK293 cells.

**FIG 1:**
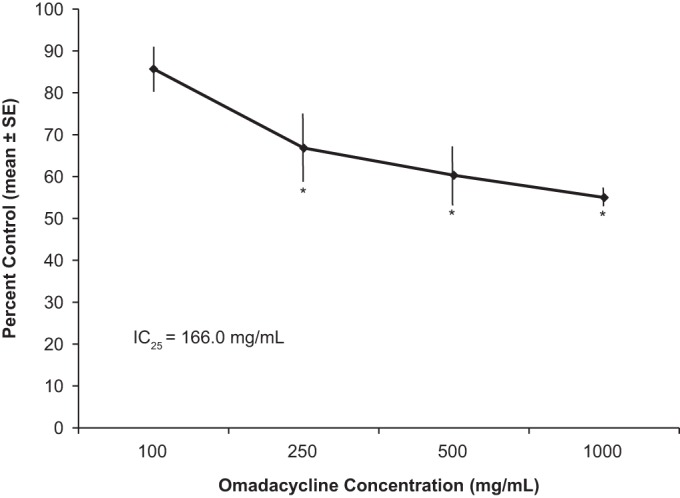
Concentration-response relationship for the effect of omadacycline on the hERG tail current. Data are corrected for mean vehicle breakdown. *, *P* < 0.01 versus the vehicle-treated group.

In vehicle-treated cells (*n* = 4), approximately 10 min of exposure to 100% bath solution produced a residual tail current of 91.1% ± 5.0% of the control values. Omadacycline at 100, 250, 500, and 1,000 μg/ml inhibited the hERG tail current in a concentration-dependent manner, and the inhibition began to plateau at higher concentrations. A significant inhibition of the tail current was observed at concentrations of 250 μg/ml and above (*P* < 0.01) compared with the results obtained with the vehicle-treated group. When the results for the omadacycline- and vehicle-treated groups were compared, omadacycline at 100 μg/ml had no significant inhibitory effect on the hERG tail current. The IC_25_ value for omadacycline was 166 μg/ml (Fig. 1). When administered to vehicle-treated cells, approximately 10 min of exposure to the K^+^ channel blocker E-4031 (100 nM; *n* = 2 cells) produced a residual tail current of 9.8% or a decrease in the tail current of 90.2%.

### *Ex vivo* sinoatrial node.

**FIG 2:**
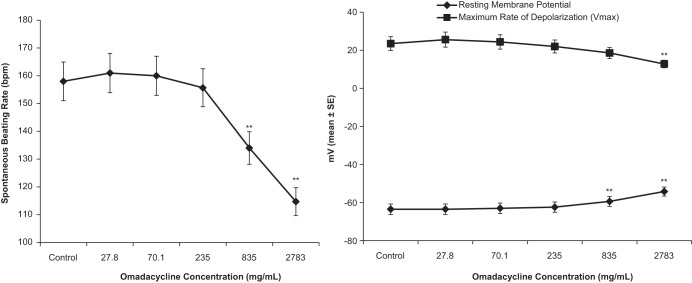
Quantitative effects of increasing omadacycline concentrations on spontaneous frequency (left) and on resting membrane potential and *V*_max_ (right) in isolated rabbit SA nodes. **, *P* < 0.01.

**FIG 3:**
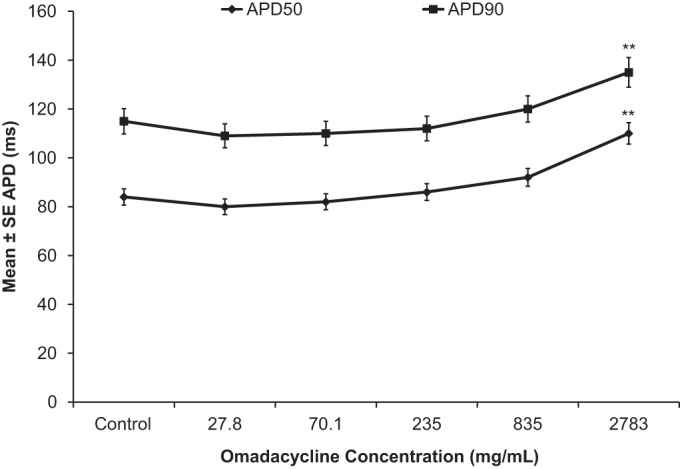
Quantitative effects of increasing omadacycline concentrations on APD_50_ and APD_90_ in isolated rabbit SA nodes. **, *P* < 0.01.

Increasing concentrations of omadacycline of 0.05, 0.126, 0.422, 1.5, and 5 mM (27.8, 70.1, 235, 835, and 2,783 μg/ml, respectively; *n* = 6 SA node preparations) had no effect on the cycle length (the time between 2 beats) or AP parameters up to a concentration of 0.422 mM (235 μg/ml). At 1.5 mM (835 μg/ml) and 5 mM (2,783 μg/ml), omadacycline markedly depolarized SA node tissue due to cytotoxicity, making the diastolic potential more positive and leading to a reduced amplitude, an increased duration, and an increased cycle length (Fig. 2). Increasing concentrations of omadacycline did not significantly modify APD_50_ or APD_90_ up to 1.5 mM (835 μg/ml) (Fig. 3). At a concentration of 2,783 μg/ml, APD_50_ and APD_90_ were significantly increased (+32% and +18%, respectively, compared with the values for the control), again likely reflecting the cytotoxicity of this concentration.

**FIG 4:**
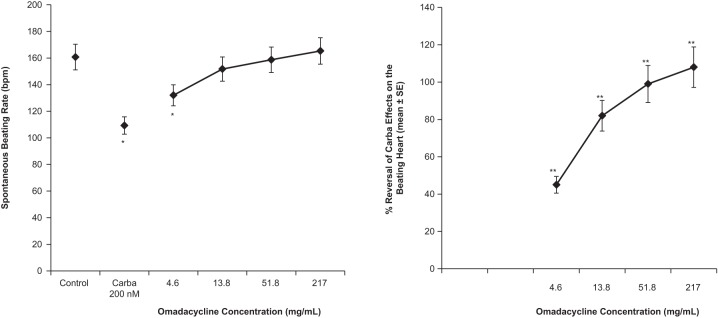
Quantitative effects of increasing omadacycline concentrations on the spontaneous beating frequency (left) and percent reversal of carbamylcholine effects (right) in isolated rabbit sinoatrial node. Carba, carbamylcholine. *, *P* < 0.01 versus the control group. The four connected symbols in each figure (from left to right, respectively) represent the results for the following treatments: 200 nM carbamylcholine and 0.008 nM (4.6 μg/ml) omadacycline, 200 nM carbamylcholine and 0.024 nM (13.8 μg/ml) omadacycline, 200 nM carbamylcholine and 0.093 nM (51.8 μg/ml) omadacycline, and 200 nM carbamylcholine and 0.39 nM (217 μg/ml) omadacycline. **, *P* < 0.01 versus the carbamylcholine-treated group.

Because omadacycline was found to inhibit the binding activity of the muscarinic M_2_ receptor, carbamylcholine (a pan-muscarinic receptor agonist) was also evaluated in these experiments. Carbamylcholine (200 nM) markedly increased the cycle length of spontaneous activity in the rabbit pacemaker SA node by decreasing the diastolic slope, leading to reduced pacemaker activity (the spontaneous beating rate was reduced by 32% ± 3% compared with the value for the control) (Fig. 4). Omadacycline reversed the effects of carbamylcholine (200 nM) on pacemaker activity. Omadacycline at 8 μM (4.6 μg/ml) significantly counterbalanced the decrease in pacemaker activity induced by carbamylcholine at 200 nM by 45% ± 4.4% (Fig. 4). This reversal was concentration dependent and reached a maximum at 93 μM (51.8 μg/ml) and 390 μM (217.1 μg/ml), which resulted in the nearly total reversal of the carbamylcholine effect (by 99% ± 9% and 108% ± 17%, respectively).

### Cardiovascular effects in conscious male cynomolgus monkeys.

**FIG 5:**
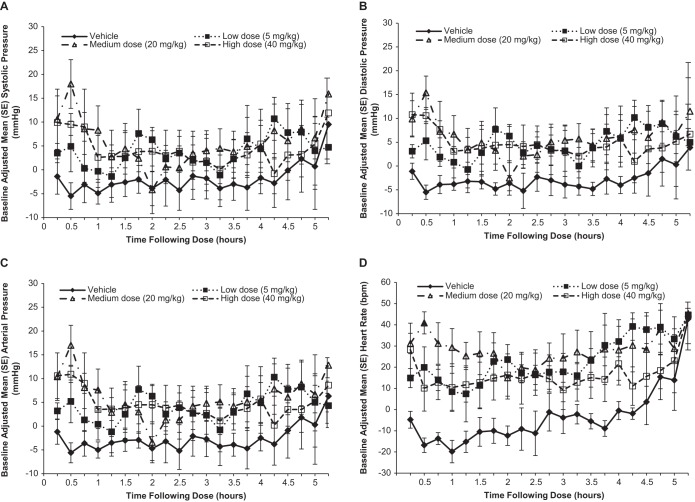
*In vivo* effects of omadacycline on monkey cardiovascular function. (A) Systolic blood pressure; (B) diastolic blood pressure; (C) mean arterial pressure; (D) heart rate. Data are the mean ± SE baseline-adjusted 15-min averages following dosing with the different doses of omadacycline or vehicle for animals in isolation boxes.

Administration of omadacycline was associated with a mildly increased arterial blood pressure (systolic, diastolic, and mean) for the 30-min interval ending at infusion termination (the time of the maximum plasma concentration [*C*_max_]) compared with that achieved with the vehicle (Fig. 5). The greatest increase was observed in the medium-dose and high-dose groups (20 and 40 mg/kg, respectively), with mean blood pressures being 23 and 17 mm Hg greater, respectively, than those in monkeys treated with the vehicle. Blood pressure trended toward baseline values after the maximum increases at 0.5 h after dose initiation. Because the animals were moved out of their isolation boxes and back to their cages after 5 h of assessment, apparent increases in blood pressure and heart rate toward the end of the assessment period in all groups (including the vehicle-treated group) may have been associated with agitation surrounding the move (which was particularly notable just following the move at hour 5.25).

Increases in heart rate were observed among the animals in all omadacycline treatment groups (Fig. 5). These changes were moderate in magnitude and were most apparent near the end of the 30-min infusion. Neither the magnitude nor the duration of heart rate changes showed a dose effect. The increase in heart rate compared with the heart rate in the vehicle-treated group was greatest (58 bpm) at 0.5 h postdosing in the group treated with an omadacycline dose of 20 mg/kg, but the increases for the groups treated with doses of 5 and 40 mg/kg were 37 and 27 bpm, respectively, compared with the heart rate for the animals in the vehicle-treated group. Compared with the animals treated with the vehicle, significant (*P* < 0.05) increases in the heart rate persisted until 4.5 h postdose for the low-dose (5 mg/kg) and medium-dose (20 mg/kg) groups and until 2.5 h postdose for the high-dose (40-mg/kg) group.

Analyses of the ECG data revealed that no significant differences in the baseline-adjusted three-beat averages for RR, PR, QT, or QTc at any omadacycline dose level compared with the results for the vehicle-treated group were observed, regardless of whether the animals were in an isolation box or in a cage. Apparent decreases in RR (the inverse of the heart rate) relative to that for the vehicle-treated group were observed, but these were not significant at the time periods when significant increases in heart rate were noted. This lack of a significant difference for RR is likely related to the different sampling methods. The RR interval is calculated over a short time interval (six beats) that is thought to be appropriate for QT correction, whereas the heart rate is represented by 15-min intervals. At 5 h following the initiation of dosing, a significant difference (*P* < 0.02) in the mean ± standard error baseline-adjusted QRS interval was observed between the vehicle-treated group (−5.3 ± 1.8 ms) and the groups receiving medium (20 mg/kg) and high (40 mg/kg) doses of omadacycline (1.3 ± 1.7 and 2.8 ± 1.7 ms, respectively). These differences appeared to reflect an increase in the heart rate (a decrease in the RR interval) in the vehicle-treated group with an associated decrease in the QRS interval. A statistically significant difference (*P* < 0.02) in the baseline-adjusted QRS between the vehicle-treated group and the group receiving the low omadacycline dose (5 mg/kg) was also observed at 24 h following the initiation of dosing (0.0 ± 2.4 and −5.0 ± 0.7 ms, respectively). These nominal QRS changes were not considered to be toxicologically meaningful or related to the study drug. For all animals, the absolute QRS intervals were within normal limits.

## DISCUSSION

*In vitro* testing indicated that omadacycline inhibits binding to a subtype of the muscarinic acetylcholine receptor (M_2_) but has no substantial effect on binding to other cholinergic receptors or other pharmacologically relevant receptor targets. In the heart, M_2_ muscarinic receptors serve as mediators of the parasympathetic input that is normally received via the vagus nerve. Stimulation of M_2_ receptors in the heart increases membrane potassium conductance through the acetylcholine-dependent channel (K_ach_), which slows depolarization and reduces pacemaker activity in the sinoatrial node (15 – 18). Other effects include a reduced conduction velocity of the atrioventricular (AV) node and reduced contractile forces of the atrial cardiac muscle; there is minimal effect on the contractile forces of the ventricular muscle (15).

The effects of inhibiting M_2_ receptor binding by omadacycline were demonstrated in the *ex vivo* rabbit sinoatrial node model. Omadacycline itself had no significant stimulatory or inhibitory effect on AP parameters or cycle length and, hence, no direct effects on the intrinsic beat rate of the SA node up to a concentration of 235 μg/ml (422 μM). However, over the concentration range of 4.6 to 217 μg/ml, omadacycline reversed the effects of the muscarinic agonist carbamylcholine in a concentration-dependent manner.

*In vivo*, following administration of omadacycline to conscious monkeys, an increase in the heart rate and a more transient increase in blood pressure parameters were observed. These were related to neither the dose nor the exposure. The changes were most apparent near the end of the omadacycline infusions, which represent the time of *C*_max_ for i.v. administration. Relative to the heart rate of the vehicle-treated control group, the increased heart rate was apparent for approximately 3 to 5 h after dosing across the dose groups of omadacycline-treated animals.

These *ex vivo* and *in vivo* findings confirm the physiological impact of the interaction of omadacycline with the M_2_ muscarinic receptor. Together, these results also suggest that the clinical effects of omadacycline may be related to the intensity of baseline parasympathetic activity (vagal tone) in any given human subject. As seen with the *ex vivo* SA node carbamylcholine experiments, in the absence of agonist binding to the M_2_ receptor, omadacycline has no direct stimulatory effects on the heart rate. These observations are consistent with the lack of omadacycline binding to any other receptors tested.

Human subjects have various degrees of vagal tone (on the basis of age and physiological, metabolic, and other factors), and it may be anticipated that the greater that the vagal tone is (and, therefore, the lower that the baseline heart rate is), the more likely it is that an increase in heart rate in response to omadacycline may be observed. To some extent, this appears to have been observed in clinical studies. In phase 1 studies in relatively young, healthy volunteers, asymptomatic increases in heart rate were observed following dosing of omadacycline (6, 19, 20). In contrast, a minimal apparent effect on heart rate was observed in a phase 2 study of omadacycline for the treatment of patients with complicated skin and skin structure infections (5). Accordingly, it can be hypothesized that in patients with active infections, the cholinergic impact on heart rate has been greatly diminished in the setting of fever, dehydration, or other systemic hypermetabolic manifestations of inflammation (thus leading to a relative tachycardia), and omadacycline will have little impact on heart rate in such a setting. Further, the increased age of patients in the phase 2 study may have reduced the vagal tone, as noted in the literature, and thus, minimal changes in heart rate would be observed (17, 21 – 23). However, this hypothesis will be further tested and analyzed in larger clinical trials in patients with serious community-acquired infections.

With respect to the important question of potential cardiac rhythm disturbances, omadacycline appeared to have a minimal, if any, effect on hERG channel activity (no effect at 100 μg/ml; IC_25_ = 166 μg/ml). The concentrations that were studied are much greater than the *C*_max_ of the typical intravenous therapeutic dose of omadacycline in humans (100 mg; *C*_max_ = 1.1 to 1.8 μg/ml) (6, 19). The *in vitro* finding is consistent with the results of the study with conscious monkeys that found no QTc changes (or other clinically significant ECG findings) with omadacycline at a dose of up to 40 mg/kg. These results suggest there is a low potential for omadacycline to have effects on the QTc in humans. This was confirmed in a subsequent thorough QTc clinical study in healthy volunteers, which found that omadacycline did not prolong QTc intervals (24).

Cardiovascular effects are not among the most common adverse events associated with tetracyclines or other antibiotics. However, some classes of antibiotics are known to possess a low but serious risk of cardiovascular toxicity related to arrhythmias (8 – 14). For example, fluoroquinolone antibiotics can prolong QTc and inhibit hERG channel function. Indeed, moxifloxacin is the standard positive-control drug in human QTc prolongation studies (7, 10, 25). Similarly, macrolides have also been known to inhibit hERG channel activity and have the potential to cause QTc prolongation (12, 13). Revised product labeling to include a warning of sudden death due to cardiac arrhythmias was required for azithromycin on the basis of reports to the U.S. Food and Drug Administration (FDA) (26). Among the tetracyclines, interaction with the M_2_ receptor appears to be a unique feature of omadacycline, at least on the basis of known adverse event profiles and accessible *in vitro* data. For example, FDA review of the antibiotic tigecycline, which is administered only by the i.v. route, found no significant binding to relevant receptors at concentrations up to 10 μM (27).

In summary, the findings from these nonclinical studies suggest that omadacycline can attenuate the parasympathetic influence on heart rate in a concentration-dependent manner. The clinical implications would be consistent with potential increases in heart rate primarily in human subjects with greater vagal tone and lower resting heart rates. The absence of any substantial β-adrenergic receptor binding coupled with the absence of any direct inhibitory or stimulatory *ex vivo* effects of omadacycline in SA node studies suggests that these heart rate increases, if observed in human subjects, should not exceed the intrinsic heart rate in the absence of vagal stimulation (a situation thus analogous to the situation in subjects who have undergone orthotopic heart transplantation, where all vagal innervation is absent). Importantly, omadacycline has a low potential for cardiovascular toxicity in human subjects related to blockade of the hERG channel. These results may assist in the interpretation of analyses of vital signs in clinical studies of omadacycline.
